# The Sanitary Organisation of the Territorial Army

**Published:** 1907-11-16

**Authors:** 


### THE SANITARY ORGANISATION OF THE TERRITORIAL ARMY.

At a meeting of the Incorporated Society of Medical Officers of Health held on November 8 Surgeon-General Sir Alfred Keogh read a paper on the "Sanitary Organisation of the New Territorial Army," now in process of formation.

"The scheme is a simple one," said Sir Alfred Keogh; "it must be simple to be practically effective."

> It has not been sufficiently recognised that the members of the medical profession were already trained in their profession, and that they were fully equipped as to knowledge of their purely professional duties. The effect of restrictions as to training in time of peace has been to exclude from participation in the national work many of the great physicians, surgeons, and especially the medical officers of health, without whom an army cannot exist.
>
> I propose, therefore, to ask the medical officers of health of the country to combine in a great voluntary organisation having for its object the preservation of the health of the large masses of men who may one day be required to defend the country. I ask them to enrol themselves in the Medical Corps of the Territorial Force.

So far as the M.O.H. himself is concerned, he is asked to contemplate his duties under the conditions of actual warfare, to expand the scope of his activities so that they shall comprehend the citizen army that is to be, and in which he will play his part in the capacity for which all his experience and training have fitted him. This, after all, is no more and no less than his present immediate duty. An army within or on the borders of his district is a "sanitary circumstance" it were short-sighted to overlook.

Only a disciplined imagination can anticipate the dispositions that would be called for; only in such provident association with military citizenship can he hope even as a civilian officer to meet the dread contingency of war.

We do not purpose here and now to elaborate the scheme sketched by Sir Alfred Keogh. In truth, its elaboration is for the future. At present it is in germ, and we can only marvel that so fresh and vigorous and attractive a growth should have germinated in so unexpected a quarter as the War Office.

